# Physical and cognitive effort discounting across different reward magnitudes: Tests of discounting models

**DOI:** 10.1371/journal.pone.0182353

**Published:** 2017-07-31

**Authors:** Wojciech Białaszek, Przemysław Marcowski, Paweł Ostaszewski

**Affiliations:** SWPS University of Social Sciences and Humanities, Warsaw, Poland; Brain and Spine Institute (ICM), FRANCE

## Abstract

The effort required to obtain a rewarding outcome is an important factor in decision-making. Describing the reward devaluation by increasing effort intensity is substantial to understanding human preferences, because every action and choice that we make is in itself effortful. To investigate how reward valuation is affected by physical and cognitive effort, we compared mathematical discounting functions derived from research on discounting. Seven discounting models were tested across three different reward magnitudes. To test the models, data were collected from a total of 114 participants recruited from the general population. For one-parameter models (hyperbolic, exponential, and parabolic), the data were explained best by the exponential model as given by a percentage of explained variance. However, after introducing an additional parameter, data obtained in the cognitive and physical effort conditions were best described by the power function model. Further analysis, using the second order Akaike and Bayesian Information Criteria, which account for model complexity, allowed us to identify the best model among all tested. We found that the power function best described the data, which corresponds to conventional analyses based on the *R*^2^ measure. This supports the conclusion that the function best describing reward devaluation by physical and cognitive effort is a concave one and is different from those that describe delay or probability discounting. In addition, consistent magnitude effects were observed that correspond to those in delay discounting research.

## Introduction

Choosing between two rewarding outcomes in day-to-day life is often not an easy feat, even more when the multiple types of costs that impact the subjective value of a particular rewarding outcome are considered. For example, a choice between smoking tobacco, which delivers immediate gratification but is associated with delayed health risks, and maintaining a more healthy lifestyle with less salient potential benefits that can occur far in time. There are multiple factors influencing such decision: delay of consequences and their probabilistic nature, and effort that is exerted to maintain our preferences. Traditional and well-documented types of factors that influence reward value include the delay until a reward can be obtained and the probability of obtaining it [1]. Substantial research from the fields of biology, neuroeconomics, behavioral economics, and psychology has been made to understand and model reward discounting by delay and probability, defined as the devaluation of a reward as the delay or uncertainty associated with obtaining that reward increases [2].

Recently, after being first introduced by Mitchell [3–4] and Sugiwaka and Okouchi [5], there has been a growing interest as to how effort-based decisions are made [6–7]. Still, the reward devaluation by effort is surprisingly understudied and is just emerging as a growing field of research. This research is important because effort exertion constitutes a prevalent factor in decision-making and the dysregulation of motivation to exert effort has been linked to disorders such as depression, abulia, apathy, or anhedonia [8–12].

Existing studies have tested the form of effort discounting. For example, Sugiwaka and Okouchi [5] tested whether rewards are devalued by effort according to a hyperbolic model that is analogous to delay or probability. This procedure required participants to indicate their preference by selecting a card representing either an effortless or an effortful hypothetical monetary payoff. After fitting the data to the hyperbolic model, the authors found that the equation accounts for 88% of the variance. No other discounting models or types of effort were tested. Similarly, Hartmann, Hager, Tobler, and Kaiser [13] examined the functional form of the discounting by physical effort. Twenty-four participants completed a computer-assisted binary-choice task that involved series of choices between effortless and effortful monetary payoffs. The authors then fitted three models to group and individual data: linear, hyperbolic, and parabolic. In the group level analysis, *R*^*2*^ values and discounting parameter estimates were obtained. For the individual model comparisons, the parameters were substituted with the group fitted parameter. The authors reported that, out of the three models, the parabolic model performed best for the group data with over 99% of variance of median subjective value accounted for. The second best model was the linear, and third best model was the hyperbolic, with 93% and 87% of variance accounted for, respectively. Individually, the three models performed similarly to groupwise analysis, with the parabolic model performing best for 18 out of 24 participants, and the linear and hyperbolic performing best for 5 and 1, respectively. Importantly, participants performed a physical effort discounting procedure only.

To date, only two studies by Ostaszewski, Bąbel, & Swebodziński [7] and Nishiyama [14] have tested model performance in both physical and cognitive effort discounting conditions. However, direct statistical comparisons of the goodness of fit indices were not provided in the study by Nishiyama [14]. In the study by Ostaszewski, Bąbel, and Swebodziński [7], participants were tasked with making series of choices between effortful and effortless alternatives of hypothetical monetary payoffs via a paper-and-pencil effort discounting questionnaire. The authors found that, for physical effort discounting, the model accounted for 87.7% of the variance in the small reward magnitude condition and 96.3% of the variance in the large reward magnitude condition. For cognitive effort discounting, the model accounted for 94.8% of the variance for small rewards and 96.6% for large rewards.

The purpose of the present study is to systematically determine whether discounting models derived from the delay discounting tradition can be applied to describe the form of effort discounting, including both physical and cognitive effort conditions with varying intensity levels, and whether a reward magnitude effect can be observed similar to that in delay discounting. A total of six models established by prior studies comprised of three models that include one free parameter and three models that include two free parameters, were fitted to the behavioral data and subsequently compared. Additionally, we have included in the comparison the power function model, based on the research by Hartmann, Hager, Tobler, and Kaiser [13], with an additional free parameter. We eliminated the possible confounding effects between effort and delay discounting within the choices made in the hypothetical scenarios by holding constant the duration of effort exertion.

First was the hyperbolic model derived from delay discounting tradition [15]:

$$SV=\frac{A}{\left(1+lE\right)},$$ (1)

where *SV* denotes the subjective value of a reward with a nominal value of *A*, receiving which requires exerting effort *E* (we substituted *D—*delay for *E* to denote effort), and *l* (*k* for *l* was substituted, which can be interpreted as the unwillingness to exert effort or a “laziness” index) is a free parameter corresponding to the steepness of the discounting curve. This model implies that additional effort has a greater impact on reward valuation in low-effort, rather than in high-effort variation. In other words, if additional effort is introduced to existing effort, it devalues a reward more than if existing effort is low rather than high.

Contrary to the first model, the second model tested, introduced by Hartmann, Hager, Tobler, & Kaiser [13] assumes that rewards are devalued parabolically with increasing effort intensity levels. That is, additional effort devalues a reward to a greater extent if existing effort is high rather than low. The parabolic model follows the equation:

$$SV=A-lE^{2}\;,$$ (2)

where *SV*, *A*, *E*, and *l* denote the same as in Eq 1. Both hyperbolic and parabolic models imply that the value of a reward is discounted to different extent across effort value spectrum, i.e., the impact of added effort is different depending on whether it is added to existing effort that is low or high; unlike the third model tested, the exponential model assumes the subjective value of a reward changes consistently across different effort values, i.e., the impact of added effort is comparable, irrespective of whether it is added to existing effort that is low or high. The exponential model was originally proposed by Samuelson [16], and includes the additional *e* denoting the Euler’s number and is the base of the natural logarithm, taking the form of:

$$SV=Ae^{-lE}\;.$$ (3)

The fourth model tested is the two-parameter hyperbola-like function, originally proposed by Myerson and Green [17], following the equation:

$$SV=\frac{A}{{\left(1+lE\right)}^{s}}\;,$$ (4)

where an additional parameter *s* is introduced, constituting the exponent of the whole denominator in the equation to reflect individual psychophysical scaling of the effect a discounting factor has in an individual, as well as that of the target reward [18]. For simplicity, we will refer to this model as Myerson and Green’s discounting model.

For the fifth model, we have tested an alternative hyperbola-like function. This model, proposed by Rachlin [2], differs from Myerson and Green’s model in that only the effort cost value is raised to a power, and not the whole denominator. In this model *s* refers to scaling of only effort intensity [18]. This model takes the following form (for simplicity, we will refer to this model as Rachlin’s discounting model):

$$SV=\frac{A}{\left(1+lE^{s}\right)}\;.$$ (5)

The sixth model compared was the two-parameter exponential model that includes additional free parameter *s*. This model was proposed by Myerson and Green [17] and takes the form of:

$$SV=\left(A-s\right)e^{-lE}+s\;,$$ (6)

where *e* denotes the same as in Eq 3 with the difference being in that including parameter *s* results in subjective value to not decay to zero as the effort increases but rather approach the asymptote of *s*.

The seventh and final model included in the comparison is a two-parameter power function. We propose this model as an extension to the conclusions from Hartmann, Hager, Tobler, and Kaiser [13], who suggest that in effort discounting the discounting curve can indeed be concave and not convex as in the case of delay or probability, with an inclusion of parameter *s* as the exponent of effort intensity to reflect individual psychophysical scaling and assure free-parameter coherence. This model takes the form of:

$$SV=A-lE^{s}\;.$$ (7)

Present study was designed to separate the cognitive and physical effort to better reflect the effort exertion effect on the valuation of a rewarding outcome in effort-based decision making. We aim to further studies such as that of Nishiyama [19]—where effort is treated as one dimension—to disambiguate results in relation to model fit comparisons. Including the two types of effort can more comprehensibly determine the functional form of effort discounting, as opposed to treating effort as one dimension, which can result in similar portion of variance explained by competing models, such as the hyperbolic and exponential [19]. We present a systematic test of a total of seven models to describe effort discounting and to determine if they can account for the functional form of discounting in both physical and cognitive effort conditions. The investigation of the mathematical form of the effort discounting asks a fundamental question, but the answer may be not so straightforward as in the case of delay or probability discounting.

## Methods and materials

We used a within-subjects design with a titration choice procedure in which all participants were exposed to each of the experimental conditions. A total of 30 conditions were included, according to the following experimental design: 2 (type of effort: physical and cognitive) x 3 (payoff amount: large, medium, and small) x 5 (effort magnitude). The order in which the conditions were presented was counterbalanced across participants.

### Participants

One hundred and fourteen participants (63 female and 59 male) between the ages of 21 to 65 (38.413 ± 9.847, mean age ± SD) were recruited for this study from the general population. The study was conducted in accordance with guidelines for ethical research approved by the Ethics Committee at the SWPS University of Social Sciences and Humanities. Each participant gave written informed consent. The participants were not compensated for participation.

### Materials

The participants were presented with a paper-and-pencil Effort Discounting Questionnaire (EDQ), adapted from previous research by Ostaszewski, Bąbel, and Swebodziński [7]. In essence, the EDQ is a behavioral procedure that is based on an approach proposed by Rachlin, Raineri, and Cross [20] and includes a fixed choice procedure with titrating value. Each experimental condition was presented on a separate page of the EDQ as series of choices between effortful and effortless alternatives of hypothetical monetary payoffs. The right column of the page contains rows of the effortful payoffs, and the left column contains rows of their corresponding effortless alternatives. The monetary value of the effortful alternatives was held constant, while the effortless alternatives were presented in descending order from 100% to 0% of their corresponding effortful counterparts in each payoff amount condition.

In the physical and cognitive effort conditions, the participants were asked to perform a number of exercises prior to the main procedure in order to become familiar with the type of effort presented in the effortful alternatives. The participants performed 20 full squeezes with a gripping device, and a total of 5 mathematical tasks, each consisting of adding three four-digit numbers in a column, for the physical and cognitive effort conditions, respectively. The experimenter ensured whether the effort was actually exerted in full.

The participants were instructed that the effortful payoff alternatives are to be received after exerting a certain magnitude of effort analogous to the preceding exercises. Payoff amounts were set to PLN 80, 400, and 3000 (1 PLN was equivalent to approximately 0.35 USD at the time of the study), for the small, medium, and large conditions, respectively. Effort magnitudes were set to 30, 60, 90, 120, and 150 instances of squeezing the gripping device or summing 3 4-digit numbers (e.g., 7812, 6352, and 2536 presented in one column), for the physical and cognitive effort conditions, respectively. During the main task, participants were asked to make choices as if they had to exert effort and as if the rewards were real; however, the main procedure did not require participants to exert real effort, and the payoffs were hypothetical.

### Procedure

Participants indicated their preference of the two payoff alternatives in each row of either the left or right column of each page: the effortless alternative (left column), in which a variable amount was to be received immediately and without effort, or the effortful alternative (right column), in which a constant amount was to be received in 30 minutes and exerting a certain amount of effort during this time. The participants indicated their preferences by circling the preferred alternative with a pencil, starting from the top row in which payoff amounts of both alternatives were equal. On each page of the EDQ, the participants made their choices until their preferences shifted from the effortless alternative to the effortful alternative, then they proceeded onto the next page of the questionnaire.

Each row took the following form: “*do you prefer to receive*: *PLN* X *now*, *without effort*, *or PLN Y after 30 minutes and exerting* Z *effort within this time*”, where X and Y were payoff values, and Z was the type of effort and its intensity. For example, in the PLN 80 and 90 squeezes physical effort condition, the topmost row was a choice between PLN 80 now and without effort, or PLN 80 after 30 minutes and performing 90 squeezes with a gripping device within this time. The monetary values of the effortless alternatives in the left column were listed as follows: 80, 79, 77, 74, 71, 68, 65, 62, 59, 56, 53, 50, 47, 44, 41, 38, 35, 32, 29, 26, 23, 20, 17, 14, 11, 8, 5, 2, 1, 0. Accordingly, in the tenth row from the top, the choice was between PLN 56 now and without effort, or PLN 80 after 30 minutes and performing 90 squeezes with a gripping device effort within this time. Such treatment was used because every effort inherently takes time to perform. We did not aim to extract the time from effort exertion and instead standardized the conditions for all participants; therefore, the time during which the effort had to be performed and simultaneously the time to reward receipt was set to a constant of 30 min.

### Measures and analysis

The experimental procedure in this study aims to identify the lowest amount of the effortless payoff to be accepted over the effortful payoff for a given effort magnitude. The lowest amount is the amount of the last effortless payoff a participant has chosen in the left column prior to switching their preference to the effortful alternative. In turn, this amount represents the subjective value of the effortful payoff for a given type and magnitude of effort, and constitutes the approximate of an indifference point (IP) in which the subjective value of the corresponding effortless and effortful alternatives is equivalent. The indifference points for each effort magnitude are then used to infer the discounting rate for each outcome amount.

For model fit and parameter comparisons, we used nonparametric tests: Friedman’s test as a general estimate of differences and Wilcoxon’s test with Sidak’s correction for multiple comparisons. To confirm the estimated parameter coherence as part of the same behavioral process, parameter correlations were tested using Spearman’s Rho coefficient. Model fitting was done using a nonlinear iterative regression approach as included in the IBM SPSS Statistics (v24). The model fit and parameters were estimated separately for the physical and cognitive effort conditions. Also, simultaneous fit across reward magnitudes was performed by fitting the equation to the data with the inclusion of 3 dummy variables paired with 3 variants of *l* and *s* parameters (one variant of each per reward magnitude) to be estimated for each model. The procedure was set in such fashion that when fitting a given model, the dummy variables sequentially set to zero other parameter variants than the one being estimated for a given reward magnitude. Effectively, this yielded a single goodness of fit measure for each model across all reward magnitudes and, at the same time, separate parameter estimates for each reward magnitude (small, medium, and large). Such approach was first outlined by Myerson and Green [17]. When the mean explained more variance than the model *R*^*2*^ was set to 0 and estimates of the parameters were set to missing values. The number of these cases were compared across models using Cochrane’s Q test, followed by multiple comparisons with the McNemar’s test.

The *l* parameter was used as a primary measure of the discounting rate. When the two parameter models were fitted to data, the second parameter was also estimated: the scaling parameter *s*. Due to time being implicitly involved in every effortful or effortless choice, the first indifference point (i.e., effortless immediate equivalent of a given reward magnitude delayed by 30 minutes) was not included in the analyses. Effort was treated as naturally occurring over time. The other reason for not including the first indifference point was that majority of subjects (71%) did not show any discounting in at least one lowest effort condition across different magnitudes of reward.

To address the complexity of models and to compare not only the models with the same number of parameters, we utilized two measures: Akaike Information Criterion (*AIC*) and Bayesian Information Criterion (*BIC*). Although *R*^*2*^ is the most traditional and widely used measure of model fit, at least in discounting research, it seems not to be the best choice for such comparisons [21–22]. As shown by Johnson and Bickel [21], the *R*^*2*^ and the parameter indicating the rate of discounting, are positively correlated, resulting in overfitting the model when the discounting is shallow. Therefore, we used additional criteria for model selection. Most important, *R*^*2*^ does not take into account the model complexity, and therefore one- and two-parameter models cannot be compared using this measure.

The *AIC* [23] lacks the weaknesses of *R*^*2*^, and has gained in popularity. *AIC* is starting to be used in discounting research [24–26]. Specifically, following guidelines outlined by Burnham and Anderson [27], we used second-order *AIC* that has an additional term for bias correction when the proportion of data points to number of parameters is low.

Although the use of this measure is growing in popularity, this is not the dominating approach, and therefore we provide some guidelines as to how we obtained an *AIC* and the goodness of model fit. The standard formula for *AIC* is:

$$AIC=n*ln\left(\frac{SSe}{n}\right)+2*p\;,$$ (8)

where *n* is number of data points (indifference points), *p* refers to the number of parameters in a model, and *SSe* is the residual (error) sum of squares obtained from nonlinear regression. The second-order *AIC* (*AIC*_*c*_) was computed as follows:

$$AIC_{c}=AIC\;+\frac{2*p*\left(p+1\right)}{n-p-1}\;.$$ (9)

Another popular measure to compare different models was proposed by Schwarz [28] and is referred to as the Bayesian Information Criterion (*BIC*).

$$BIC=n*ln\left(\frac{SSe}{n}\right)+\text{ln}\left(n\right)*p$$ (10)

As can be seen, the second-order *AIC* has an additional penalty for model complexity added to the first-order *AIC*. The lower the value of *AIC*_*c*_ and *BIC*, the better the model [26–31]. We used relative rather than absolute *AIC*_*c*_ and *BIC* values, computed as the difference between *AIC*_*c*_ (or *BIC*) of a given model and *AIC*_*c*_ (or *BIC*) of the model with the best fit (lowest value) of all compared models. This resulted in a best model having the delta *AIC*_*c*_ = 0 (or *BIC* = 0) for a single-participant or a single-group comparison. Again, the lower the delta values for a model, the better the model fit. We performed all comparisons on the median group level (the median was computed from individual indifference points) and on summed *AIC*_*c*_ and *BIC* values across subjects as advised by Peters, Miedl, & Büchel [26]. Such aggregation of values of information criteria across subjects would correspond to fixed-effect analyses. On the other hand, a random-effect analysis in Bayesian Model Selection (BMS) treats models as random variables that can differ between subjects (for a description and comparison of Group Bayes Factor (GBF) approaches and BMS as linked to fixed and random effect analysis, see Stephan et al. [32]). The *BIC* differs from *AIC* by the additional penalty parameter that it gives to models of different complexity. Rank-wise, *BIC* and *AIC* give the same result when models of the same complexity are compared. Therefore, we decided to address the issue of overall model comparison in a separate section of our paper at the end of the results section.

## Results

First, we fitted one-parameter Eqs 1, 2 and 3 to the appropriate group median indifference points (cognitive and physical effort, separately), simultaneously to three different amount conditions, yielding three separate *l* parameters for each equation and three separate estimates of goodness of fit (*R*^*2*^, one for all magnitude conditions). Second, we analogously fitted the two-parameter models 4, 5, 6, and 7 to the data, yielding three separate *l* parameters and three separate *s* parameters. Third, to be able to identify the single best model, which is impossible when using only *R*^*2*^ measure, we used *AIC*_*c*_ and *BIC* that account for model complexity. This allowed for parameter comparisons between cognitive and physical effort.

### One-parameter models in cognitive effort

In the cognitive effort conditions, there was a statistically significant difference between three model fits (*χ*^*2*^ = 30.67; *p*< .001). The exponential model obtained the highest mean rank (*M*_*rank*_ = 2.38) and the hyperbolic model obtained the middle mean rank (*M*_*rank*_ = 1.94). The parabolic model obtained the lowest rank (*M*_*rank*_ = 1.68). All differences between models were statistically significant. The exponential model described the individual data significantly better 68.4% and tied 6.1% of the time (78 and 7 cases, respectively) compared to the hyperbolic model (*Z* = 4.56; *p*< .001; *r* = .30) that performed significantly better 63.2% and tied 7% of the time (72 and 8 cases, respectively) when compared to the parabolic model (*Z* = 4.69; *p*< .001; *r* = .31). The hyperbolic model explained more variance than the parabolic model (*Z* = 4.21; *p*< .001; *r* = .28) 62.3% and tied 6.1% of the time (71 and 7 cases, respectively). The median and interquartile range for the *R*^*2*^ and parameter values for one-parameter models in the cognitive effort condition are presented in Table 1.

**Table 1:** Median and interquartile range for *R*^*2*^ and *l* parameters from three one-parameter models, fitted to data on group median (i.e., fit to median IP) and individual level in cognitive effort conditions.

Cognitive effort												
	Hyperbolic				Exponential				Parabolic			
	*R*^2^	*l*_*80*_	*l*_*400*_	*l*_*3000*_	*R*^2^	*l*_*80*_	*l*_*400*_	*l*_*3000*_	*R*^2^	*l*_*80*_	*l*_*400*_	*l*_*3000*_
Fit to median IP	0.86	4.96E-03	4.04E-03	2.56E-05	0.89	4.04E-03	2.34E-03	9.37E-04	0.95	2.56E-05	1.66E-05	7.62E-06
Median	0.70	6.76E-03	3.43E-03	1.75E-03	0.72	5.05E-03	2.74E-03	1.58E-03	0.59	2.36E-05	1.40E-05	3.97E-06
25^th^	0.51	1.99E-03	1.38E-03	1.65E-04	0.56	1.80E-03	1.25E-03	1.58E-04	0.00	1.01E-05	6.03E-06	5.67E-07
75^th^	0.88	2.16E-02	1.03E-02	6.69E-03	0.90	1.27E-02	7.34E-03	4.73E-03	0.80	5.19E-05	3.19E-05	2.36E-05
*R*^*2*^ = 0*	7				8				33			

*This corresponds to the mean explaining more variance than the model.

All differences between *l* parameters in the three reward magnitudes in cognitive effort conditions were highly significant (*p*< .001 in all cases). For convenience, we report only the results from the exponential model, however all other differences between *l* parameters in regard to hyperbolic or parabolic models were highly significant (*p<* .001). There were significant differences between discounting parameters (*χ*^*2*^ = 74.92; p< .001). The mean ranks decreased as the amount increased (2.53; 2.11; 2.36). As expected, the *l* parameter for PLN 80 was largest, and significantly different from *l* parameter for PLN 80 and PLN 400 (*Z* = 5.23; *p*< .001; *r* = .35), for PLN 400 and PLN 3000 (*Z* = 5.78; *p*< .001; *r* = .38) and for PLN 80 and PLN 300 (*Z* = 6.78; *p*< .001; *r* = .45).

To confirm that *l* parameters estimated across different reward magnitude conditions refer to the same behavioral process, there was a significant correlation between *l* parameters across the three reward magnitudes. For the highest reward magnitude of PLN 3000, the parameter correlated significantly with *l* for PLN 400 condition (*r*_*s*_ = .77; p< .001) and parameter *l* for PLN 80 condition (*r*_*s*_ = .58; p< .001); there was also a significant correlation between parameter *l* for PLN 400 and 80 conditions (*r*_*s*_ = .69; p< .001).

Despite the goodness of fit estimates given by proportion of explained variance, the number of cases in which the mean explained more variance than the model is also of importance (i.e., treated as *R*^*2*^ = 0). We found there are significant differences in the number of cases in which the mean explained more variance than the model (*χ*^*2*^ = 50.077; *p*< .001). The best fitted exponential model did not fit the data in 8 cases, while the hyperbolic and parabolic models did not fit the data in 7 and 33 cases, respectively. Following pairwise comparisons revealed that the bestfitted exponential model only differed in the number in cases that did not fit data with the parabolic model (*χ*^*2*^ = 23.040; *p*< .001), while the numbers of such cases for the exponential and hyperbolic models were comparable (*p* = 1.000).

### One-parameter models in physical effort

**Fig 1:**
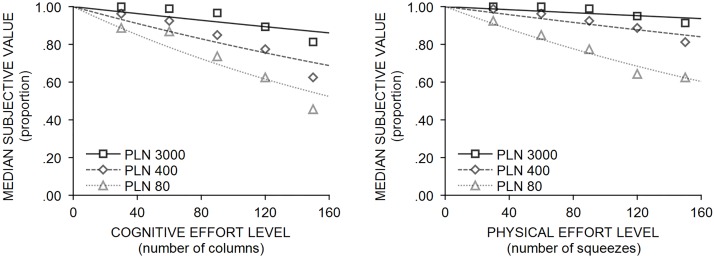
Empirical indifference points obtained in cognitive (left panel) and physical (right panel) effort discounting, with curves fitted corresponding to the one-parameter exponential model in three different reward magnitudes.

We observed the same pattern of differences between *R*^*2*^ values in physical effort as in cognitive effort conditions. There are significant differences between model fits (*χ* ^*2*^ = 27.72; p< .001). The mean ranks of exponential, hyperbolic, and parabolic models are respectively: 2.33, 1.98, and 1.58. Table 2 displays the median and interquartile range for the *R*^*2*^ and parameter estimates for one-parameter models in the physical effort condition. Indifference points with curves fitted that correspond to the best fitted one-parameter exponential model in three different reward magnitudes (in cognitive and physical effort condition) are illustrated in Fig 1.

**Table 2:** Median and interquartile range for *R*^2^ and *l* parameters from three one-parameter models, fitted to data on group median (i.e., fit to median IP) and individual level in physical effort conditions.

Physical effort												
	Hyperbolic				Exponential				Parabolic			
	*R*^2^	*l*_*80*_	*l*_*400*_	*l*_*3000*_	*R*^2^	*l*_*80*_	*l*_*400*_	*l*_*3000*_	*R*^2^	*l*_*80*_	*l*_*400*_	*l*_*3000*_
Fit to median IP	0.95	3.77E-03	3.16E-03	2.02E-05	0.96	3.16E-03	1.09E-03	4.10E-04	0.88	2.02E-05	8.31E-06	3.47E-06
Median	0.69	6.41E-03	2.41E-03	9.89E-04	0.74	4.73E-03	1.98E-03	9.59E-04	0.55	1.11E-05	5.07E-06	2.28E-05
25^th^	0.52	1.40E-03	4.25E-04	5.48E-05	0.50	1.30E-03	4.17E-04	5.47E-05	0.00	2.58E-06	2.84E-07	9.24E-06
75^th^	0.85	2.24E-02	1.01E-02	7.63E-03	0.87	1.28E-02	7.21E-03	5.77E-03	0.80	3.50E-05	2.62E-05	4.96E-05
*R*^*2*^ = 0*	14				15				24			

*This corresponds to the mean explaining more variance than the model.

The exponential model described the individual data significantly better 60.5% and tied 13.2% of the time (69 and 15 cases, respectively) in comparison to the hyperbolic model (*Z* = 3.67; *p*< .001; *r* = .24); performed better 59.6% and tied 13.2% of the time (68 and 15 cases, respectively) compared to the parabolic model (*Z* = 5.45; *p*< .001; *r* = .36). The hyperbolic model explained more variance than the parabolic model (*Z* = 4.77; *p*< .001; *r* = .32) 58.8% and tied 13.2% of the time (67 and 15 cases, respectively).

Likewise, in relation to parameter *l*, the same pattern was observed as in the cognitive effort conditions. There were significant differences between *l* parameters estimated by the exponential model (*χ* ^*2*^ = 88.51; *p*< .001). These differences were representative for all three models (*p<* .001). The mean ranks decreased as the amount increased (2.72, 1.89, and 1.39), and these differences were statistically significant, i.e., between PLN 80 and PLN 400 (*Z* = 6.19; *p*< .001; *r* = .41), PLN 400 and PLN 3000 (*Z* = 4.16; *p*< .001; *r* = .28) and between PLN 80 and PLN 300 (*Z* = 6.78; *p*< .001; *r* = .45).

Similar to the physical effort condition, *l* parameters were significantly correlated across three reward magnitudes. Parameter *l* for the highest reward magnitude of PLN 3000 correlated significantly with parameter *l* for PLN 400 condition (*r*_*s*_ = .76; *p*< .001) and with parameter *l* for PLN 80 condition (*r*_*s*_ = .56; *p*< .001); the correlation between parameter *l* for PLN 400 and 80 conditions was also significant (*r*_*s*_ = .77; *p*< .001).

Again, there was a significant difference in the number of cases in which data did not fit the model (*χ*^*2*^ = 30.000; *p*< .001). The best fitted exponential model and the hyperbolic model did not fit the data in 15 cases, while the parabolic model did not fit the data in 30 cases. Accordingly, for the exponential model, the difference in the number of those cases was only significant between the parabolic (*χ*^*2*^ = 13.067; *p*< .001), and not the hyperbolic model (*p* = 1.000).

### Two-parameter models in cognitive effort

For cognitive effort, we observed a statistically significant difference between the four two-parameter model fits (*χ*^*2*^ = 100.926; *p*< .001). The highest mean rank was obtained by the power function model (*M*_*rank*_ = 3.29), followed by the exponential model (*M*_*rank*_ = 2.55), the Rachlin’s model (*M*_*rank*_ = 2.52), and finally the Myerson and Green’s model (*M*_*rank*_ = 1.63). The power function model described the individual data significantly better 69.3% and tied 5.3% of the time (79 and 6 cases, respectively) when compared to the exponential model (*Z* = 5.272; *p*< .001; *r* = .47). The power function model performed better 69.3% and tied 5.3% of the time (79 and 6 cases, respectively) when compared to Rachlin’s model (*Z* = 5.036; *p*< .001; *r* = .33); and performed better 82.5% and tied 5.3% of the time (94 and 6 cases, respectively) in comparison to Myerson and Green’s model (*Z* = 7.451; *p*< .001; *r* = .49) (refer to Table 3 for median and interquartile range for the *R*^*2*^ and parameter estimates for two-parameter models in cognitive effort condition).

**Table 3:** Median and interquartile range for the *R*^2^ and *l* and *s* parameters from three two-parameter models, fitted to the data on group median (i.e., fit to median IP) and individual level in cognitive effort conditions.

Cognitive effort						
		Fit to median IP	Median	25^th^	75^th^	*R*^*2*^ = 0*
Myerson & Green	*R*^*2*^	0.89	0.80	0.61	0.92	7
	*l*_*80*_	4.06E-04	9.70E-04	4.38E-04	2.44E-02	
	*s*_*80*_	1.00E+01	6.75E+00	7.38E-01	2.24E+01	
	*l*_*400*_	6.71E-04	1.09E-03	3.49E-04	7.68E-03	
	*s*_*400*_	3.55E+00	1.88E+00	3.83E-01	1.62E+01	
	*l*_*3000*_	5.24E-04	4.77E-04	2.05E-04	1.12E-03	
	*s*_*3000*_	1.82E+00	1.41E+00	4.03E-01	9.84E+00	
Rachlin	*R*^*2*^	0.96	0.82	0.00	0.93	30
	*l*_*80*_	3.89E-05	1.20E-04	4.97E-06	6.68E-03	
	*s*_*80*_	2.04E+00	2.30E+00	1.23E+00	3.10E+00	
	*l*_*400*_	2.45E-05	1.68E-04	3.80E-06	3.60E-03	
	*s*_*400*_	2.00E+00	1.78E+00	9.06E-01	2.75E+00	
	*l*_*3000*_	1.19E-04	2.61E-04	4.57E-06	3.51E-03	
	*s*_*3000*_	1.45E+00	1.49E+00	6.17E-01	2.48E+00	
Exponential	*R*^*2*^	0.94	0.85	0.74	0.94	6
	*l*_*80*_	1.56E-03	3.90E-03	7.89E-04	2.45E-02	
	*s*_*80*_	-1.27E+00	-1.36E-01	-2.79E+00	2.82E-01	
	*l*_*400*_	1.34E-03	2.27E-02	1.46E-03	9.97E-02	
	*s*_*400*_	-1.18E+00	1.42E-01	-8.69E-01	7.91E-01	
	*l*_*3000*_	7.32E-04	8.02E-02	5.42E-03	5.40E-01	
	*s*_*3000*_	-1.19E+00	7.50E-01	-9.92E-02	9.85E-01	
Power function	*R*^*2*^	0.99	0.93	0.81	0.97	8
	*l*_*80*_	6.14E-04	8.29E-03	3.38E-04	1.01E-01	
	*s*_*80*_	1.35E+00	7.48E-01	3.07E-01	1.31E+00	
	*l*_*400*_	4.73E-05	2.62E-03	1.23E-05	4.74E-02	
	*s*_*400*_	1.79E+00	8.50E-01	4.17E-01	1.85E+00	
	*l*_*3000*_	6.22E-08	1.21E-04	4.70E-07	1.04E-02	
	*s*_*3000*_	2.98E+00	1.17E+00	5.16E-01	2.59E+00	

*This corresponds to the mean explaining more variance than the model.

We then compared *l* and *s* parameter values across the three reward magnitudes within the best-fitted power function model. For *l*, there was a significant difference between PLN 80, 400, and 3000 conditions (*χ*^*2*^ = 35.133; *p*< .001). The value of the *l* parameter was highest for the lowest reward magnitude condition (PLN 80), and then decreased as the reward magnitude increased with *M*_*rank*_ = 2.35, *M*_*rank*_ = 2.08, and *M*_*rank*_ = 1.57 for the PLN 80, 400, and 3000 reward conditions, respectively. Further comparisons revealed that the highest *l* parameter value, obtained in the PLN 80 condition, differed significantly from *l* obtained for the PLN 400 (*Z* = 2.961; *p* = .003; *r* = .20) and PLN 3000 (*Z* = 4.538; *p*< .001; *r* = .31) reward conditions. The difference in *l* for the PLN 3000 and PLN 400 conditions was also significant (*Z* = 3.547; *p*< .001; *r* = .23).

As for the *s* parameter, the differences across the three reward magnitudes were overall significant (*χ*^*2*^ = 10.892; *p* = .004). There was an increase in the value of *s* that corresponded with the increase in reward magnitudes (mean ranks: 1.77, 2.01, 2.22 for PLN 80, 400, and 3000, respectively). Parameter *s* differed significantly between PLN 80 and PLN 400 (*Z* = 2.606; *p* = .009; *r* = .18), and between PLN 80 and PLN 3000 (*Z* = 3.173; *p* = .002; *r* = .22) reward magnitude conditions; however, the difference between the PLN 3000 and PLN 400 conditions did not reach statistical significance (*p* = .140).

A similar pattern of correlation across the three reward magnitudes was observed for parameters *l* and *s* as for one-parameter models. In the cognitive effort condition, parameter *l* obtained for the highest reward magnitude of PLN 3000 was significantly correlated with *l* obtained for PLN 400 (*r*_*s*_ = .69; *p*< .001) and with *l* obtained for PLN 80 (*r*_*s*_ = .53; *p*< .001). Parameter *l* obtained for PLN 400 also significantly correlated with *l* for PLN 80 (*r*_*s*_ = .56; p< .001). Likewise, parameter *s* obtained for PLN 3000 was correlated between *s* obtained for PLN 400 (*r*_*s*_ = .57; *p*< .001) and between *s* for PLN 80 (*r*_*s*_ = .47; *p*< .001). There was also a significant correlation between parameter *s* for PLN 400 and parameter *s* for PLN 80 (*r*_*s*_ = .53; p< .001)

The cases in which each model did not fit the data at a satisfactory level were individually verified. There was an overall difference in the number of cases for which *R*^*2*^ = 0 (*χ*^*2*^ = 60.570; *p*< .001). The best fitted power function model did not fit the data in 8 cases, while the models proposed by Rachlin, Myerson and Green, and the exponential model did not fit the data in 30, 7, and 6 cases, respectively. Still, pairwise comparisons with the McNemar test showed that the best fitted power function model only differed in the number of cases that did not fit the data with the Rachlin’s model (*χ*^*2*^ = 16.962; *p*< .001), while the differences with the exponential model and the Myerson and Green’s model did not reach significance (*p* = .480 and *p* = 1.000, for the exponential and Myerson and Green’s model, respectively).

### Two-parameter models in physical effort

**Fig 2:**
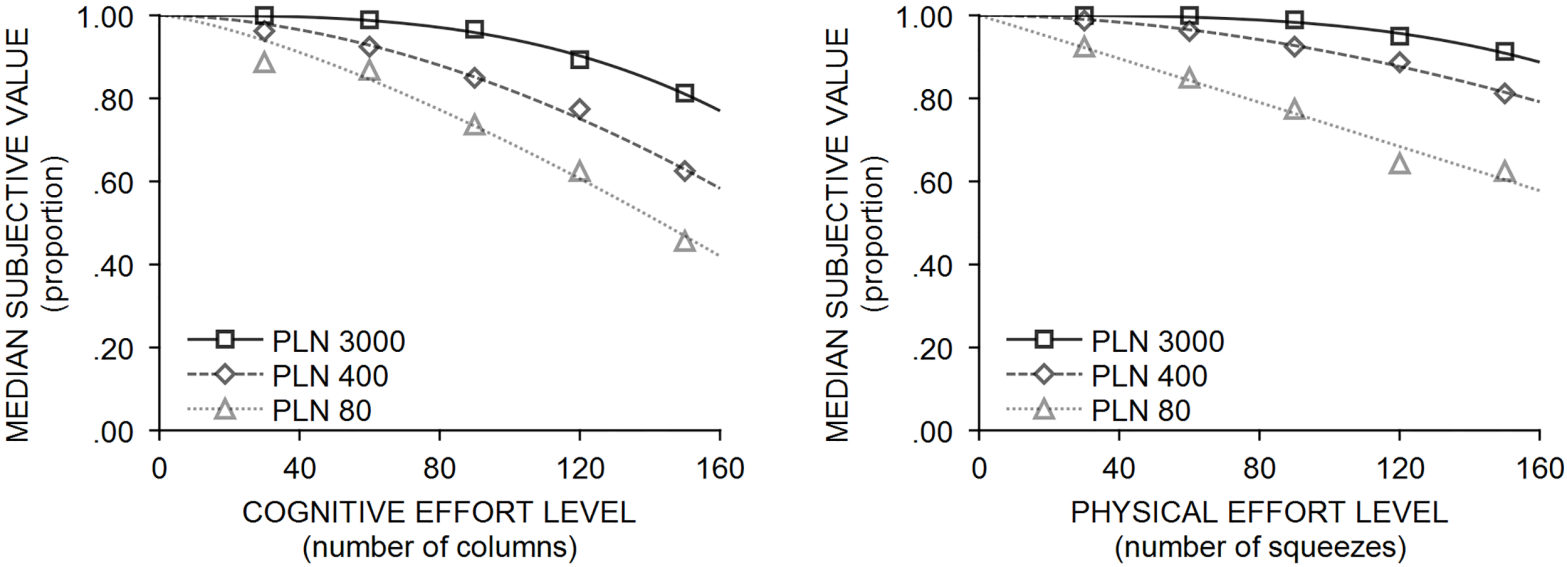
Empirical indifference points obtained in cognitive (left panel) and physical (right panel) effort discounting, with curves fitted corresponding to the two-parameter power function model in three different reward magnitudes.

For physical effort, similar to the cognitive effort condition, there was a significant difference in models fits across two-parameter models (*χ*^*2*^ = 123.195; *p*< .001). Again, the highest mean rank was obtained by the power function model (*M*_*rank*_ = 3.47), followed by the exponential model (*M*_*rank*_ = 2.48), and finally, the models proposed by Rachlin (*M*_*rank*_ = 2.36) and Myerson and Green (*M*_*rank*_ = 1.70). At the individual level, the power function model described the data better 69.3% and tied 5.3% of the time (79 and 6 cases, respectively) compared to the exponential model (*Z* = 5.272; *p*< .001; *r* = .35), better 71.1% and tied 10.5% of the time (81 and 12 cases, respectively) compared to Rachlin’s model (*Z* = 6.274; *p*< .001; *r* = .42), and was better 84.2% and tied 10.5% of the time (96 and 12 cases respectively) when compared to Myerson and Green’s model (*Z* = 7.923; *p*< .001; *r* = .52). Table 4 presents the median and interquartile range for the *R*^*2*^ and parameter estimates from three two-parameter models in physical effort conditions. Illustrated in Fig 2 are the indifference points and curves fitted from the best-fitted two-parameter power function model in three different reward magnitudes in the cognitive and physical effort conditions.

**Table 4:** Median and interquartile range for *R*^*2*^ and *l* and *s* parameters from three two-parameter models, fitted to data on group median (i.e., fit to median IP) and individual level in physical effort conditions.

Physical effort						
		Fit to median IP	Median	25^th^	75^th^	*R*^*2*^ = 0*
Myerson & Green	*R*^*2*^	0.96	0.80	0.63	0.90	13
	*l*_*80*_	7.40E-04	1.14E-03	3.86E-04	6.51E-02	
	*s*_*80*_	4.44E+00	4.04E+00	4.02E-01	1.39E+01	
	*l*_*400*_	8.01E-04	6.84E-04	3.43E-04	7.47E-03	
	*s*_*400*_	1.42E+00	1.35E+00	2.21E-01	1.04E+01	
	*l*_*3000*_	1.87E-03	3.60E-04	7.01E-05	1.25E-03	
	*s*_*3000*_	2.38E-01	1.31E+00	2.63E-01	9.35E+00	
Rachlin	*R*^*2*^	0.97	0.81	0.00	0.91	41
	*l*_*80*_	2.58E-04	1.25E-03	6.78E-06	3.77E-02	
	*s*_*80*_	1.57E+00	1.64E+00	7.61E-01	3.14E+00	
	*l*_*400*_	1.17E-04	3.12E-04	1.43E-05	1.01E-02	
	*s*_*400*_	1.48E+00	1.42E+00	5.29E-01	2.35E+00	
	*l*_*3000*_	1.59E-04	2.99E-05	1.67E-06	2.15E-03	
	*s*_*3000*_	1.21E+00	1.49E+00	7.03E-01	2.63E+00	
Exponential	*R*^*2*^	0.96	0.86	0.74	0.92	12
	*l*_*80*_	1.63E-03	2.71E-03	7.68E-04	3.21E-02	
	*s*_*80*_	-7.88E-01	-1.01E-01	-2.30E+00	5.04E-01	
	*l*_*400*_	8.50E-04	4.52E-02	5.34E-03	1.60E-01	
	*s*_*400*_	-7.41E-01	4.77E-01	-1.26E-01	9.49E-01	
	*l*_*3000*_	1.28E-01	9.56E-02	6.32E-03	6.20E-01	
	*s*_*3000*_	9.63E-01	8.38E-01	-4.89E-02	9.95E-01	
Power function	*R*^*2*^	0.99	0.92	0.82	0.97	12
	*l*_*80*_	2.51E-03	1.09E-02	2.72E-04	1.19E-01	
	*s*_*80*_	1.01E+00	7.39E-01	1.97E-01	1.31E+00	
	*l*_*400*_	1.93E-05	1.73E-03	6.03E-07	1.89E-02	
	*s*_*400*_	1.83E+00	8.45E-01	5.12E-01	1.90E+00	
	*l*_*3000*_	5.71E-09	1.65E-05	6.34E-11	2.64E-03	
	*s*_*3000*_	3.31E+00	1.24E+00	5.61E-01	2.65E+00	

*This corresponds to the mean explaining more variance than the model.

For the *l* parameter values computed for the best-fitted power function model across the three reward magnitudes, there was an overall difference across PLN 80, 400, and 3,000 conditions (*χ*^*2*^ = 57.210; *p*< .001). Value of the *l* parameter was highest for the lowest reward magnitude condition (PLN 80) and then decreased as the reward magnitude increased (*M*_*rank*_ = 2.55, *M*_*rank*_ = 1.93, and *M*_*rank*_ = 1.52 for the PLN 80, 400, and 3000 reward conditions, respectively). Pairwise comparisons show that the highest *l* parameter obtained for the PLN 80 condition differed significantly from *l* obtained for the PLN 400 (*Z* = 3.925; *p*< .001; *r* = .26) and from *l* obtained for the PLN 3000 reward condition (*Z* = 6.191; *p*< .001; *r* = .41); the difference in *l* for the PLN 3000 and PLN 400 conditions was also statistically significant (*Z* = 2.467; *p* = .014; *r* = .16).

For the *s* parameter values computed for the best-fitted power function model across all reward magnitudes, there was an overall difference between PLN 80, 400, and 3000 conditions (*χ*^*2*^ = 19.317; *p*< .001). There was an increase in *s* parameter value that corresponds with the increase in reward magnitudes with mean ranks: 1.66, 2.11, and 2.24 for PLN 80, 400, and 3000 reward conditions. Further comparisons showed that the highest parameter *s* value obtained for the highest reward magnitude (PLN 80) differed significantly between the PLN 400 (*Z* = 3.244; *p* = .001; *r* = .21) and PLN 3000 (*Z* = 4.865; *p*< .001) conditions; the difference in *s* parameters for the PLN 3000 and PLN 400 reward magnitudes was likewise significant.

As in the cognitive effort condition, there was a significant correlation observed for both *l* and *s* parameters across three reward magnitudes in the physical effort condition. Parameter *l* obtained for the highest reward magnitude of PLN 3000 was correlated with *l* obtained for PLN 400 (*r*_*s*_ = .58; *p*< .001) and *l* obtained for PLN 80 (*r*_*s*_ = .43; *p*< .001). Also, a correlation between *l* for PLN 400 and 80 was observed (*r*_*s*_ = .48; *p*< .001). Parameter *s* obtained for PLN 3000 correlated with *s* obtained for PLN 400 (*r*_*s*_ = .47; *p*< .001) and between *s* for PLN 80 (*r*_*s*_ = .42; *p*< .001). Parameter *s* for PLN 400 likewise correlated with *s* for PLN 80 (*r*_*s*_ = .41; *p*< .001).

We verified the number of cases for which the models did not fit the data at a satisfactory level, there was an overall difference in the number of cases for which *R*^*2*^ = 0 across all models (*χ*^*2*^ = 84.136; *p*< .001). Best fitted power function model did not fit the data in 12 cases, while the exponential, Rachlin’s, and Myerson and Green’s models did not fit the data in 12, 41, and 13 cases, respectively. Likewise to the cognitive effort condition, further comparisons showed that the best fitted power function models differed in the number of non-fit cases only with the Rachlin’s model (*χ*^*2*^ = 27.034; *p*< .001), while the differences between the exponential model and the Myerson and Green’s model did not reach significance (*p* = 1.000 and *p* = 1.000 for the exponential model and Myerson and Green’s model; these significance levels equal 1 because of the correction for multiple comparisons).

### Model comparisons accounting for model complexity

Previous measures and comparisons did not allow for a comparison between different models. To identify the best model among the seven tested, we used measures such as *AIC*_*c*_ and *BIC* that take into account model complexity and allow for comparisons between models with one and two free parameters. We performed model comparisons on the group level, i.e., fitting the models to median group-level indifference points, and on the individual level, computing the *AIC*_*c*_ and *BIC* for every participant.

On the median group level, the best-fitting model in the physical effort conditions, as determined by *AIC*_*c*_, was the exponential model. However, the power function and hyperbolic model were plausible in yielding a delta *AIC* below 2 [27, 31]. The *BIC* criterion determined that the power function model was best in this situation. For the cognitive effort, the *AIC*_*c*_ determined the parabolic function was the best fitting, and again there was substantial support for the power function but not the exponential model. The *BIC* consistently showed the power function as the best model solution. The delta values of both criteria are presented in Table 5.

**Table 5:** Model comparisons using *AIC*_*c*_ and *BIC* criteria on median group level (data were fitted to median indifference points) and aggregate. Summed across participants *AIC*_*c*_ and *BIC*, values were converted to delta values.

	One-parameter models			Two-parameter models			
Group							
Physical	Hyperbolic	Exponential	Parabolic	Myerson & Green	Rachlin	Exponential	Power function
ΔAICc	1.33	0	5.61	7.17	4.69	6.72	0.89
ΔBIC	5.5	4.17	9.78	6.28	3.8	5.83	0
Cognitive							
ΔAICc	5.61	4.16	0	11.1	6	7.49	0.47
ΔBIC	10.19	8.74	4.58	10.63	5.52	7.02	0
Aggregate							
Physical	Hyperbolic	Exponential	Parabolic	Myerson & Green	Rachlin	Exponential	Power function
ΔAICc	962.93	960.64	1090.18	1182.48	1650.13	2855.81	0
ΔBIC	1539.57	1538.88	1666.77	1182.42	1650.1	2943.9	0
Cognitive							
ΔAICc	547.53	431.44	754.51	936.56	1172.27	1992.97	0
ΔBIC	1124.12	1007.95	1331.04	936.59	1172.25	2054	0

The choice of median to infer the group-level model performance is only one possible solutions in the majority of studies on discounting. As we mentioned in the measures and analysis section, we also analyzed the differences between the best-fitting model and a given model on summed *AIC*_*c*_ and *BIC* values across subjects. These results are listed in Table 5. These analyses uniformly (regardless of criterion used) pointed to the power function model as best describing the behavior on the group level, yielding the 0 value for this model.

Overall, examining the delta *AIC*_*c*_ and *BIC* measures for the seven models in physical and cognitive effort conditions from two group-level perspectives, we conclude that the power function had the strongest support, giving the values of both criteria below 2 in all situations and yielding the values of delta = 0 (Table 5). As can be seen from Table 5, *AIC*_*c*_ penalized more complex models a bit more than did *BIC*, but this is a natural relationship for these measures when the number of data points is small, and reverses for a large number of points [31].

To further support the conclusions drawn from the median group-level comparisons, we tested the model performance on an individual level. This time, however, we focused on the frequencies in cases where the given model was the best model (Table 6).

**Table 6:** Model comparisons on individual level. Table contains frequencies and ratios of cases for which the given model yielded best fit.

	One-parameter models			Two-parameter models			
	Hyperbolic	Exponential	Parabolic	Myerson & Green	Rachlin	Exponential	Power function
Physical							
ΔAICc	19 (16.7%)	31 (27.2%)	35 (30.7%)	0	4 (3.5%)	2 (1.8%)	23 (20.2%)
ΔBIC	6 (5.3%)	11 (9.6%)	14 (12.3%)	0	14 (12.3%)	6 (5.3%)	64 (56.1%)
Cognitive							
ΔAICc	17 (14.9%)	33 (28.9%)	34 (29.8%)	0	6 (5.3%)	3 (2.6%)	21 (18.4%)
ΔBIC	5 (4.4%)	5(4.4%)	16 (14.0%)	0	16 (14.0%)	12 (10.5%)	60 (52.6%)

In general, an examination of Table 6 indicates some discrepancies between *AIC*_*c*_ and *BIC*. The first measure pointed to the one-parameter solution, with the simple parabola being the most frequent model. The *AIC*_*c*_ identified the parabolic model as best in 35 cases in physical-effort conditions, and in 34 cases in cognitive-effort conditions, with the exponential model having close estimations frequency-wise. By contrast, the *BIC* measure pointed very clearly to the power function as being best in the description of individual data.

Considering the group-level data, which pointed overall to the power function as being best across different conditions, and to individual-level analyses based on both the *AIC*_*c*_ and *BIC*, we conclude that in the situation of effortful decision-making, our data suggests that the power function best describes a participant’s choices.

Our final comparisons focused on the relation between physical and cognitive effort discounting. We found that overall there are moderate correlations between *l* parameters reflecting different rates of amount-dependent discounting between physical and cognitive effort conditions (Table 7). All of these correlations were significant.

**Table 7:** Correlations (Spearman’s rho coefficient) for power function model *l*, and *s* parameters for cognitive and physical effort conditions across three reward magnitudes.

	physical *l*_PLN 80_	physical *l*_PLN 400_	physical *l*_PLN 3000_	cognitive *l*_PLN 80_	cognitive *l*_PLN 400_
physical *l*_PLN 400_	.484**				
physical *l*_PLN 3000_	.426**	.582**			
cognitive *l*_PLN 80_	.525**	.377**	.229*		
cognitive *l*_PLN 400_	.397**	.473**	.326**	.560**	
cognitive *l*_PLN 3000_	.367**	.506**	.426**	.533**	.694**

*significant at *p*< .05;

**significant at *p*< .01.

Similar to the *s* parameters, all correlations were significant and varied from weak to moderate (Table 8), and all of them were positive.

**Table 8:** Correlations (Spearman’s rho coefficient) of power-function model *s* parameters for cognitive and physical effort conditions across three reward magnitudes.

	physical *s*_80_	physical *s*_400_	physical *s*_3000_	cognitive *s*_80_	cognitive *s*_400_
physical *s*_PLN 400_	.410**				
physical *s*_PLN 3000_	.418**	.471**			
cognitive *s*_PLN 80_	.439**	.265*	.123		
cognitive *s*_PLN 400_	.350**	.423**	.283*	.527**	
cognitive *s*_PLN 3000_	.288*	.234*	.309*	.473**	.570**

*significant at *p*< .05;

**significant at *p*< .01.

As for the correlations of the l and *s* parameters for the power-function model fitted to data in physical and cognitive effort conditions, we observed negative relationships ranging from weak to strong (Table 9). These corresponded to the inverse effect of the reward magnitude on the two parameters observed in estimate comparisons.

**Table 9:** Correlations (Spearman’s rho coefficient) between power-function model *l* and *s* parameters for physical and cognitive effort conditions across three reward magnitudes.

	Physical effort			Cognitive effort		
	*s*_80_	*s*_400_	*s*_3000_	*s*_80_	*s*_400_	*s*_3000_
*l*_80_	-.953	-.331	-.383	-.923	-.521	-.436
*l*_400_	-.466	-.804	-.379	-.458	-.902	-.529
*l*_3000_	-.376	-.382	-.550	-.451	-.606	-.760

*Note*: all correlations significant at *p*< .01

## Discussion

The primary aim of this paper was to investigate cognitive and physical effort discounting functions derived from the delay discounting tradition and to show whether the magnitude effect is present in effort discounting. Unlike in the domain of delay or probability discounting [2], we found that the hyperbolic or hyperboloid function was not superior in describing the data in effort discounting. When we considered only one-parameter models, the exponential function had the overall best fit. However, in the two free-parameter solutions, the power function explained the highest proportion of the variance. When we controlled for model complexity by using *AIC*_*c*_ and *BIC*, we found that when considering both group-level model fit and model fit on an individual level, the power function described the data better than the remaining models. In addition, we presented a replication of the magnitude effect, well documented in delay discounting research and also shown in effort discounting. Sensitivity to effort manifested most vividly in the small reward value conditions. As the reward magnitude increased, participants seemed to be less and less sensitive to increases in effort intensity.

There is a large discrepancy between research results on effort discounting. Regardless of the character of effort (real or hypothetical) research shows that the underlying model of effort discounting can be hyperbolic [7, 14] or parabolic [13]. Additionally, some research suggests that there is no dominant model in this type of discounting [19]. In line with our results, research by Klein-Flügge et al. [33] also points out that the shape of effort discounting function might not be convex, but concave, contrary to delay or probability discounting. A convex function tends to underestimate reward value for lower effort levels, compared to a concave function, which tends to overestimate reward value for higher effort levels. This is supported by our results for one-parameter and two-parameter models. For two-parameter models, when examining the amount of explained variance, a concave power function model obtained the best fit. For one-parameter models, the reason why *R*^2^ pointed to exponential model as best, may be that this model by definition overestimates the reward value in lower effort values and underestimates it in higher effort values. This corresponds to the results obtained for two-parameter models because the concave two-parameter power function behaves similarly at low and high values of the discounting factor.

Therefore, we conclude that both physical and cognitive effort devalues rewards less in lower effort values and more in higher effort values, i.e., in low effort variation, the discounting curve is less steep and devalued more when additional effort is added to existing effort that is high rather than low, i.e., in high effort variation, the discounting curve is more steep.

This concave form of reward devaluation by effort (cognitive and physical) can, in part, be supported by the relative low effect of low energy costs in humans, as discussed by Klein-Flügge et al. [33]. Therefore, single instances of nondemanding effortful tasks, such as those in the present study, have limited impact on subjective reward value. Authors further suggest that it is when effort costs exceed one’s individual threshold of sensitivity, i.e., when effort costs starts being perceived as taxing, reward value is discounted to a greater extent. Although this seems in line with our results, we argue to further differentiate types of effortful tasks into: (1) repeated effort tasks, in which single instances of effort exerted accumulate over time (e.g., multiple muscle contractions or attention switching), where effort intensity is the number of contractions; and (2) tasks that consist of sustained effort exertion (e.g., single sustained muscle contraction or maintaining attention), where effort intensity is the percentage of maximum voluntary effort sustained for a set time period. The nature of the cognitive and physical effort tasks in present study was that their single instances were associated with relative low energy costs. Further research could focus on comparing reward devaluation in different types of tasks. Second, it would be interesting to investigate whether preferences toward a given type of effort further impact the form it devalues rewards. Considering preferences not only in terms of cognitive or physical effort but also with the inclusion of preferences toward different tasks (preferred or non-preferred) within the domains of cognitive or physical effort would be valuable. Furthermore, psychophysiological predispositions towards specific forms of effort should also be considered. Third, the observed low sensitivity to effort, evidenced by low discounting rates observed in higher reward magnitudes, might have been supported by the low demand of a single effort unit within our procedure, conjointly with the largely simulated nature of effort used. We propose that further research determine if similar observations to ours can be made with other types of effort tasks performed within both physical and cognitive effort domains. Last but not least, the abovementioned directions would be well-applied in further research addressing effort-based choice behavior in loss situations, as first introduced by Nishiyama [14].

Another support for the concave power function comes from the effort characteristics themselves and theoretical considerations. As pointed by Le Bouc et al. [34], convex functions previously usually tested in delay discounting (in our study this refers to: hyperbolic, exponential, or hyperboloid) do converge asymptotically to 0 with an increase in effort, but they do not reach the actual value of 0. This would correspond to an observation that even an extremely delayed reward is better compared to receiving nothing immediately. Unlike these models, concave functions can reach the value of 0, or even as Le Bouc et al. [34] show in their subtractive discounting approach, also negative values are possible. Conversely, approaches that allow the value function to reach 0 (e.g., the power function) would account for a situation when the effort needed to obtain a reward exceeds individual capabilities. We find this notion interesting from the standpoint of behavioral economics because it would signify that, unlike in delay discounting, the principle of rational choice is inapplicable. Accordingly, for example, no matter how great the incentive of a reward, a high enough effort cost would make that reward unattainable. Consistently with considerations from Le Bouc et al. [34], we therefore concur that the asymptotic functions do not appear to be appropriate for the effort domain.

In other words, no matter how great the delay to reward receipt, waiting would always be preferable to choosing nothing at all over an (even greatly) delayed reward. This is not so when the reward value is discounted as a function of effort, because here, choosing nothing over a reward (no matter how large of a magnitude) that is contingent on effort costs that exceed one’s capabilities is conceivable. Therefore, we believe that approaches that allow the function to reach 0 can account for such situations, while those that approach 0 asymptotically cannot. There are of course other possible models that could be tested, for example, the sigmoidal model derived by Klein-Flügge et al. [33]. The sigmoidal function decreases asymptotically when effort requirements become increasingly high. In our opinion, it is possible that approaches such as the power function may result in negative subjective values that are more valid in the situation of devaluation by effort. It is possible that the exceedingly high costs of effort would not only yield a choice that points to accepting nothing instead of exerting effort, but would also yield a negative value, i.e., participants would be prone to pay not to engage in a given activity. This, however, would require another methodological approach that would accommodate possible negative subjective values expressed as losses.

**Fig 3:**
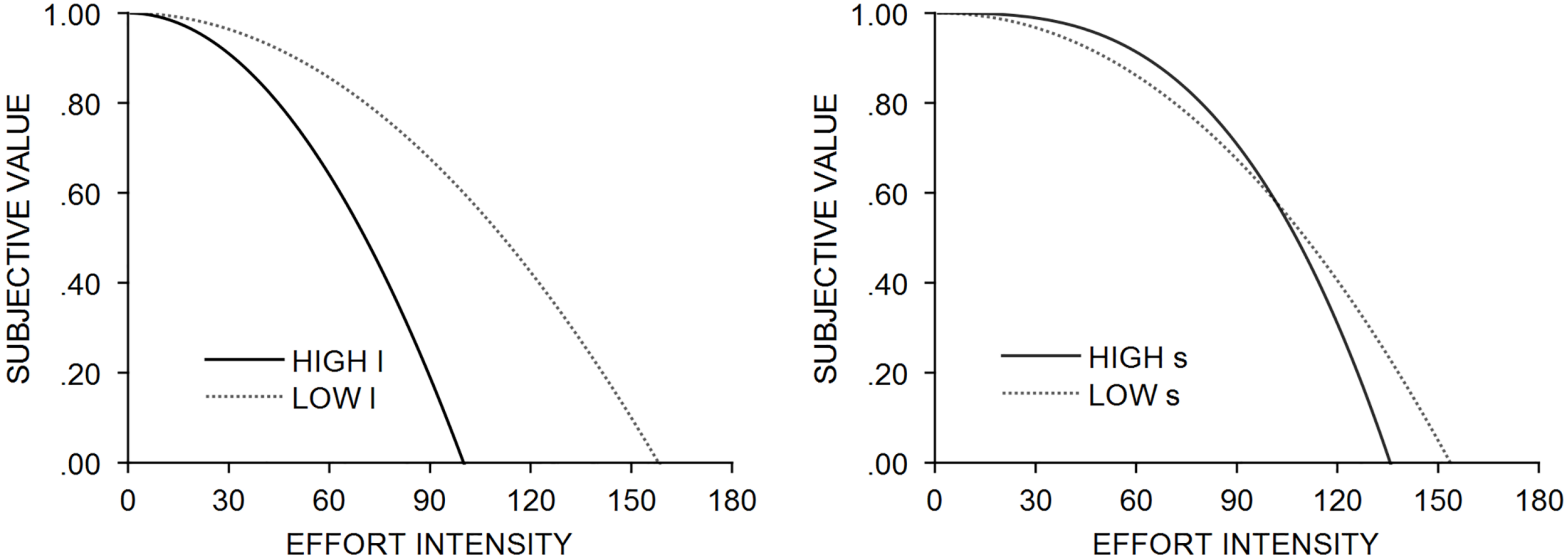
Theoretical account for the effects of varying parameter *s* and *l* values: Holding constant the value of *s* with low and high *l* values (left panel), and holding constant the value of *l* with low and high *s* values (right panel).

Our next observation refers to discounting parameters *l* and *s*. When the power function exponent parameter takes the value of *s* = 1, subjective reward value devalues in a linear fashion, with parameter *l* determining the slope of the function. With an upward increase in power from *s* = 1, the function becomes more concave with holding the *l* parameter constant. In the theoretical situation illustrated in Fig 3, we observe that adding the free power exponent to parabolic discounting function (which now becomes a power function), results in the function becoming flatter in the low effort values, and decreases more rapidly in the higher effort values. Specifically, if parameter *l* is held constant at values estimated empirically for each reward magnitude, increasing the value of *s* parameter results in the function being less steep in lower effort values and more steep in higher effort values, contrary to the opposite situation with *l* held constant and decreasing the value of *s* resulting in the function being more steep in lower effort values and more steep in higher effort values. If parameter *s* is held constant, changing the value of *l* from low to high results in an overall increase of the discounting rate, i.e., a more steep discounting curve and more rapid reward devaluation with increasing effort intensity. Here, again the best fit for the one-parameter exponential model, indicated by *R*^2^, supports even stronger our findings for two-parameter models in such fashion that higher values of *s* result in the discounting curve being less steep in low effort values and more steep in higher effort values. The one-parameter exponential model behaves in similar way as compared to the two-parameter power function model: it overestimates reward value in low range of a discounting factor and underestimates value in high range of a discounting factor.

Our second main finding is that the devaluation of rewards is amount-dependent. In both conditions, i.e., the physical and cognitive discounting, we observed similar patterns of amount dependency. Specifically, large monetary gains were discounted proportionally less than smaller payoffs. The direction of the magnitude effect and its presence is in line with the observation that large rewards have greater motivational power. For example, animal research on rats showed that rats run faster when a large reward is the outcome [35–36]. Results in human studies showed mixed results.

As reviewed by [37], many studies indicate that an increase in amount of the reward increases the performance, some show no decrease in performance, and a minority of the studies show a decrease in performance. The presence of the magnitude effect may provide some theoretical ground for interpretation of such nonuniform conclusions. The performance of a given task, according to our study, is amount dependent. In that sense, the magnitude of reward sometimes has a larger and sometimes a smaller impact on performance. For small-reward conditions where the discounting is steeper, in low-effort conditions an additional requirement or task will decrease the performance more than in comparable changes when the stakes are high. This is owing to the concave shape of the function. However, when the reward for exerting effort is large, the performance changes only slightly in initial effort values, but when the effort is higher, it drops more rapidly according to a power function shape. Some research suggests that the presence of the magnitude effect may be another characteristic of populations with impulse-control problems [38].

Interestingly, the power function *s* parameter estimates for the PLN 80 and 400 reward magnitudes are inferior to 1 (Tables 3 and 4), suggesting a convex discounting, whereas estimates superior to 1 would be expected in a concave power function (Fig 2). This is reflected in the function shape in such a fashion that a continued decrease of *s* below 1 when holding *l* constant results in a progressively more shallow convex discounting. This might be a consequence of an asymmetric distribution of the parameter estimates. Effectively, due to its nature, the median in skewed distributions is less influenced by the extreme values. In our data, mean values for all parameters are always greater than 1. Because the *s* distributions were positively skewed, the median in respect to the mean is shifted towards lower values. Therefore, we aggregated the *AIC* and *BIC* measures to represent group-level analyses (Table 5), following the approach outlined by Peters et al. [39].

With regard to the relation of *l* and *s* parameters, we observed a double effect of the reward magnitude, i.e., with increasing reward magnitude, parameter *l* values decreased while the values of *s* increased. For l, this observation could be considered as indicating that cost perception is relative to the anticipated benefit. Therefore, the ratio of cost to benefit could account for the magnitude effect, as previous modeling approaches suggest that reward valuation in physical [40], cognitive [41], or both effort domains [42] can be captured by a cost-benefit trade-off. In those approaches, effort costs are described by a parabolic function. For physical effort cost, the function shape can be particularly more marked compared to a power function: flat for low-effort values and more pronounced for high-effort values [34].

With these considerations, the magnitude effect observed on the *s* parameter might be an artifact of using a less-pronounced discount function. Alternatively, with research from Rachlin [2] and McKerchar et al. [18], if we interpret the *l* parameter as the index of unwillingness to exert effort, then its effect should diminish as the reward incentive increases with larger reward magnitudes. If the *s* parameter corresponds to psychophysical stimuli scaling, then the scaling can refer to the perception of reward magnitude or effort cost or both. If the scaling is an effect of both effort costs and reward magnitude [17, 43], it could be that the parameter *s* estimates and the shape of the value function for different reward magnitudes in low and high effort (i.e., flat for low effort and more pronounced for high effort) is a mixed product of the (potentially) different sensitivities to effort costs and reward incentive. Given the present results, and that the effort discounting can be linked to pathological reward processing, further investigation of magnitude-related phenomena is needed.

We also observed significant correlations between physical and cognitive effort discounting. The same direction of the effect of amount on effort discounting in physical and cognitive effort conditions, combined with a finding that both types of effort correlate, provide support for the hypothesis that the extent to which effortful costs impact our decisions has trait-like characteristics (see Tables 7 and 8 for correlations of *l* and *s* parameters between effort domains and across reward magnitudes). Although it is very plausible, it needs further investigation, especially when using real or quasi-real rewards. In addition, moderate relationships between reward devaluation by physical and cognitive effort may reflect partially overlapping and partially distinct neural mechanisms that underlie cost-benefit analyses involving different effort domains [44]. Support for the positive correlation of physical activity and cognitive abilities comes from numerous studies showing the positive relationship of engaging in physical activity and general cognitive performance, or the weakening of cognitive decline in certain populations [45–47]. On the other hand, a recent study by Chong et al. [44] revealed that there was no significant relationship between cognitive and physical effort discounting parameters, reflecting the discounting rate. This result, which differs from ours, can be task specific and needs systematic replication.

By identifying the best model, we were able to make comparisons between physical- and cognitive effort discounting rates. We found weak to moderate correlations of *l* and *s* between physical and cognitive effort discounting and across reward magnitudes (Table 9). We believe this reflects the overlapping of neurocomputational mechanisms that underlie the estimation of reward-cost trade-offs, as well as separate metabolic and psychophysiological bases of the two effort domains [48]. In other words, if *l* corresponds to the unwillingness to perform effort and *s* to the psychophysical scaling of effort costs, parameter correlations may indicate that the parameters refer to the same processes of cost-benefit analysis in physical and cognitive effort conditions but are contributed to by different resources and biological mediators.

One of the problems with effort as a decision cost is that, unlike delay or probability, it is not unidimensional. To address the delay confounding in effortful tasks, we included equal task performance time in the data (effectively including performance time in the measurement of the indifference points) that allowed control of the confounding effects of delay inherently required to perform a task. This is because every effortful task requires time to perform. Although by constraining the delay of the delivery of the reward, we rule out possible changes in the time of effort exertion, such approach bears some limitations. For example, it should be noted that the paper-and-pencil discounting task formulation might overestimatethe rate of effort discounting, because reward devaluation is elicited in part also by the fixed delay to reward receipt. On the other hand, as mentioned in the methods section, the majority of subjects did not discount at all during effortless conditions introducing only the 30 minute delay. Also, in these conditions, indifference points medians were equal to the nominal values of rewards. In other words, it seems that the delay to reward receipt had negligible impact on the indifference points. However, further research may address the possible interactions of explicitly two-dimensional costs of time and effort required to obtain a given outcome.

Furthermore, time is inherent to behavior performance and every action takes time. Although effort and delay share some common characterictics, devaluation of rewards by effort or time is described by apparently different functions. This seems consistent with prior reports suggesting that the effect of effort and delay on reward devaluation might differ on behavioral and brain level. For example, Klein-Flügge et al. [33] reported dissociable behavioral effects of physical effort and delay in reward devaluation. In conjunction, a study by Massar et al. [49] suggested that separate as well as overlapping neural substrates encode reward valuation in delay and effort discounting. In addition, Prevost et al. [50] arrived at similar conclusions, showing that even though on a behavioral level, delay and physical effort discounting of erotic stimuli might seem as similar processes, distinct neural valuation subsystems were responsible for reward devaluation. Conclusions from these studies are also supported by Malesza and Ostaszewski [51], who showed similar correlational patterns between effort and delay discounting in relation to temperamental variables.

A parallel between delay and effort discounting might manifests itself in the presence of the reward magnitude effect. Further studies can focus on whether a relation of reward amount and discounting rate holds for tasks in which effort duration is extracted. Already some experimental paradigms intentionally limit or control for duration of effort exertion, either by implementing quasi-instantaneous actions, presenting only options with constant durations (for example: [10,34,44,50]), or increasing the time at which the effortless reward is received by the average time required to perform the effort that corresponds to the effortful reward [52]. This presents an interesting question for further investigations: if and how reward devaluation results might differ if obtained through experimental procedures that either extract effort performance duration or approach effort as naturally occurring over time (with all its characteristics)? As both approaches seem interesting, comparing the results obtained by each might stimulate further valuable discussion.

Along with these conclusions, we demonstrate that, in physical and cognitive effort discounting, both parameter *l* and s were amount dependent, unlike in delay discounting [17], where parameter *s* is amount independent. It should be noted that these results are not directly comparable because of the different nature of the models. Myerson and Green’s model is hyperboloid, and our two-parameter equation is a power function.

In our study, we took two paths of analysis. First, we compared models using the widely used *R*^*2*^ measure. These analyses pointed to two possible models: the exponential model as best among one-parameter models, and the power function as best among two-parameter models. Because this approach was not conclusive, we decided to utilize the information criteria to account for model complexity.

We found that on the group and individual levels, the two-parameter power function described the data better than the alternative models. With regard to the models that we tested, the intention of our work was to compare prominent models from the discounting tradition to test their applicability in effort discounting. This was done in order to determine if models investigated in other forms of discounting (in particular, delay discounting) would also describe how rewards are devalued as a function of effort costs.

Of course, there are numerous other approaches to the modeling of such choices. For example, other theories such as reinforcement learning [40], or those suggesting a different function shape, e.g., a sigmoid [33] or Le Bouc et al. [34], tested primarily the physical effort. In addition, it is possible to incorporate, in further studies, inferences based on Bayesian Model Selection (BMS) procedures such as those described by Daunizeau, Adam, and Rigoux, and Rigoux et al. [53–54]. These account for accuracy and model complexity.

We decided to use the most common criteria (*AIC* and *BIC*) used in previous studies on discounting, but we are aware of the fact that other model selection criteria can be used (e.g., free energy [55]). Therefore, our intention was to not use all known models and approaches, but to maintain coherence with the line of works on discounting within behavioral economics. Despite still growing interest in the investigation of how effort impacts reward valuation, it is still largely unclear how it drives individual preferences. Further advancement of research on effort-based preferences will contribute to better understanding how effort exertion impacts the valuation of rewarding outcomes of our choices and provide guidelines for behavioral programs aimed at supporting recovery in much of the prevalent motivational and mood disorders, linked to the diminished willingness to exert effort.

## Supporting information

S1 File Effort data. Raw data from the study along with fit indices.
(SAV)

## References

[1] Green L, Myerson J. A Discounting Framework for Choice With Delayed and Probabilistic Rewards. Psychological Bulletin. 2004;130(5):769–792. doi: 10.1037/0033-2909.130.5.769 15367080 10.1037/0033-2909.130.5.769 PMC1382186

[2] Rachlin H. Notes on Discounting. Journal of the Experimental Analysis of Behavior. 2006;85(3):425–435. 16776060 10.1901/jeab.2006.85-05 PMC1459845

[3] Mitchell S. Measures of impulsivity in cigarette smokers and non-smokers. Psychopharmacology. 1999;146(4):455–464. 10550496 10.1007/pl00005491

[4] Mitchell S. Effects of short-term nicotine deprivation on decision-making: Delay, uncertainty and effort discounting. Nicotine & Tobacco Research. 2004;6(5):819–828. 15700917 10.1080/14622200412331296002

[5] Sugiwaka H, Okouchi H. Reformative self-control and discounting of reward value by delay or effort1. Japanese Psychological Research. 2004;46(1):1–9.

[6] Botvinick M, Huffstetler S, McGuire J. Effort discounting in human nucleus accumbens. Cognitive, Affective, & Behavioral Neuroscience. 2009;9(1):16–27. 10.3758/CABN.9.1.16 PMC2744387 19246324

[7] Ostaszewski P, Bąbel P, Swebodziński B. Physical and cognitive effort discounting of hypothetical monetary rewards. Japanese Psychological Research. 2013; 55(4):329–337.

[8] Cummings J. Anatomic and Behavioral Aspects of Frontal-Subcortical Circuits. Annals of the New York Academy of Sciences. 1995;769(1):1–14. 10.1111/j.1749-6632.1995.tb38127.x 8595019

[9] Treadway M, Buckholtz J, Schwartzman A, Lambert W, Zald D. Worth the ‘EEfRT’? The Effort Expenditure for Rewards Task as an Objective Measure of Motivation and Anhedonia. PLoS ONE. 2009;4(8):e6598 doi: 10.1371/journal.pone.0006598 19672310 10.1371/journal.pone.0006598 PMC2720457

[10] Cléry-Melin M, Schmidt L, Lafargue G, Baup N, Fossati P, Pessiglione M. Why Don't You Try Harder? An Investigation of Effort Production in Major Depression. PLoS ONE. 2011;6(8):e23178 doi: 10.1371/journal.pone.0023178 21853083 10.1371/journal.pone.0023178 PMC3154289

[11] Treadway M, Bossaller N, Shelton R, Zald D. Effort-based decision-making in major depressive disorder: A translational model of motivational anhedonia. Journal of Abnormal Psychology. 2012;121(3):553–558. doi: 10.1037/a0028813 22775583 10.1037/a0028813 PMC3730492

[12] Hartmann M, Hager O, Reimann A, Chumbley J, Kirschner M, Seifritz E et al Apathy But Not Diminished Expression in Schizophrenia Is Associated With Discounting of Monetary Rewards by Physical Effort. Schizophrenia Bulletin. 2014;41(2):503–512. doi: 10.1093/schbul/sbu102 25053653 10.1093/schbul/sbu102 PMC4332944

[13] Hartmann M, Hager O, Tobler P, Kaiser S. Parabolic discounting of monetary rewards by physical effort. Behavioural Processes. 2013;100:192–196. doi: 10.1016/j.beproc.2013.09.014 24140077 10.1016/j.beproc.2013.09.014

[14] Nishiyama R. Physical, emotional, and cognitive effort discounting in gain and loss situations. Behavioural Processes. 2016;125:72–75. doi: 10.1016/j.beproc.2016.02.004 26876919 10.1016/j.beproc.2016.02.004

[15] Mazur J. An adjusting procedure for studying delayed reinforcement In: Commons M, Mazur J, Nevin J, ed. by. The Effect of Delay and of Intervening Events on Reinforcement Value: Quantitative Analyses of Behavior, Volume V 1st ed United Kingdom: Taylor and Francis; 2013 p. 55–73.

[16] Samuelson P. A Note on Measurement of Utility. The Review of Economic Studies. 1937;4(2):155.

[17] Myerson J, Green L. Discounting of delayed rewards: Models of individual choice. Journal of the Experimental Analysis of Behavior. 1995;64(3):263–276. doi: 10.1901/jeab.1995.64-263 16812772 10.1901/jeab.1995.64-263 PMC1350137

[18] McKerchar T, Green L, Myerson J, Pickford T, Hill J, Stout S. A comparison of four models of delay discounting in humans. Behavioural Processes. 2009;81(2):256–259. doi: 10.1016/j.beproc.2008.12.017 19150645 10.1016/j.beproc.2008.12.017 PMC2674118

[19] Nishiyama R. Response effort discounts the subjective value of rewards. Behavioural Processes. 2014;107:175–177. doi: 10.1016/j.beproc.2014.08.002 25150069 10.1016/j.beproc.2014.08.002

[20] Rachlin H, Raineri A, Cross D. Subjective probability and delay. Journal of the Experimental Analysis of Behavior. 1991;55(2):233–244. doi: 10.1901/jeab.1991.55-233 2037827 10.1901/jeab.1991.55-233 PMC1323057

[21] Johnson M, Bickel W. An algorithm for identifying nonsystematic delay-discounting data. Experimental and Clinical Psychopharmacology. 2008;16(3):264–274. doi: 10.1037/1064-1297.16.3.264 18540786 10.1037/1064-1297.16.3.264 PMC2765051

[22] Spiess A, Neumeyer N. An evaluation of R2 as an inadequate measure for nonlinear models in pharmacological and biochemical research: a Monte Carlo approach. BMC Pharmacology. 2010;10(1):6. 20529254 10.1186/1471-2210-10-6 PMC2892436

[23] Akaike H. A new look at the statistical model identification. IEEE Transactions on Automatic Control. 1974;19(6):716–723.

[24] Hayashi Y, Miller K, Foreman A, Wirth O. A behavioral economic analysis of texting while driving: Delay discounting processes. Accident Analysis & Prevention. 2016;97:132–140. 27614547 10.1016/j.aap.2016.08.028 PMC5154926

[25] Mitchell S, Wilson V, Karalunas S. Comparing hyperbolic, delay-amount sensitivity and present-bias models of delay discounting. Behavioural Processes. 2015;114:52–62. doi: 10.1016/j.beproc.2015.03.006 25796454 10.1016/j.beproc.2015.03.006 PMC4404224

[26] Peters J, Miedl S, Büchel C. Formal Comparison of Dual-Parameter Temporal Discounting Models in Controls and Pathological Gamblers. PLoS ONE. 2012;7(11):e47225 doi: 10.1371/journal.pone.0047225 23226198 10.1371/journal.pone.0047225 PMC3511467

[27] Burnham K, Anderson D. Model Selection and Multimodel Inference: A Practical Information-Theoretic Approach. New York: Springer-Verlag; 2002.

[28] Schwarz G. Estimating the Dimension of a Model. The Annals of Statistics. 1978;6(2):461–464.

[29] Neath A, Cavanaugh J. The Bayesian information criterion: background, derivation, and applications. Wiley Interdisciplinary Reviews: Computational Statistics. 2011;4(2):199–203.

[30] Kass R, Raftery A. Bayes Factors. Journal of the American Statistical Association. 1995;90(430):773–795.

[31] Wagenmakers E, Farrell S. AIC model selection using Akaike weights. Psychonomic Bulletin & Review. 2004;11(1):192–196. 15117008 10.3758/bf03206482

[32] Stephan KE, Penny WD, Daunizeau J, Moran RJ, Friston KJ. Bayesian model selection for group studies. Neuroimage. 2009;46(4):1004–17. Available from: http://dx.doi.org/10.1016/j.neuroimage.2009.03.025 19306932 10.1016/j.neuroimage.2009.03.025 PMC2703732

[33] Klein-Flügge M, Kennerley S, Saraiva A, Penny W, Bestmann S. Behavioral Modeling of Human Choices Reveals Dissociable Effects of Physical Effort and Temporal Delay on Reward Devaluation. PLOS Computational Biology. 2015;11(3):e1004116 doi: 10.1371/journal.pcbi.1004116 25816114 10.1371/journal.pcbi.1004116 PMC4376637

[34] Le Bouc R, Rigoux L, Schmidt L, Degos B, Welter M, Vidailhet M et al Computational Dissection of Dopamine Motor and Motivational Functions in Humans. Journal of Neuroscience. 2016;36(25):6623–6633. doi: 10.1523/JNEUROSCI.3078-15.2016 27335396 10.1523/JNEUROSCI.3078-15.2016 PMC6601748

[35] Crespi L. Quantitative Variation of Incentive and Performance in the White Rat. The American Journal of Psychology. 1942;55(4):467.

[36] Mellgren R. Positive and negative contrast effects using delayed reinforcement. Learning and Motivation, 1972, 3 2: 185–193.

[37] Bonner S, Sprinkle G. The effects of monetary incentives on effort and task performance: theories, evidence, and a framework for research. Accounting, Organizations and Society. 2002;27(4–5):303–345.

[38] Mellis A, Woodford A, Stein J, Bickel W. A second type of magnitude effect: Reinforcer magnitude differentiates delay discounting between substance users and controls. Journal of the Experimental Analysis of Behavior. 2017;107(1):151–160. doi: 10.1002/jeab.235 28101922 10.1002/jeab.235 PMC5321101

[39] Peters J, Miedl S, Büchel C. Formal Comparison of Dual-Parameter Temporal Discounting Models in Controls and Pathological Gamblers. PLoS ONE. 2012;7(11):e47225 doi: 10.1371/journal.pone.0047225 23226198 10.1371/journal.pone.0047225 PMC3511467

[40] Rigoux L, Guigon E. A Model of Reward- and Effort-Based Optimal Decision Making and Motor Control. PLoS Computational Biology. 2012;8(10):e1002716 doi: 10.1371/journal.pcbi.1002716 23055916 10.1371/journal.pcbi.1002716 PMC3464194

[41] Shenhav A, Botvinick M, Cohen J. The Expected Value of Control: An Integrative Theory of Anterior Cingulate Cortex Function. Neuron. 2013;79(2):217–240. doi: 10.1016/j.neuron.2013.07.007 23889930 10.1016/j.neuron.2013.07.007 PMC3767969

[42] Manohar S, Chong T, Apps M, Batla A, Stamelou M, Jarman P et al Reward Pays the Cost of Noise Reduction in Motor and Cognitive Control. Current Biology. 2015;25(13):1707–1716. doi: 10.1016/j.cub.2015.05.038 26096975 10.1016/j.cub.2015.05.038 PMC4557747

[43] Green L, Fry A, Myerson J. Discounting of Delayed Rewards: A Life-Span Comparison. Psychological Science. 1994;5(1):33–36.

[44] Chong T, Apps M, Giehl K, Sillence A, Grima L, Husain M. Neurocomputational mechanisms underlying subjective valuation of effort costs. PLOS Biology. 2017;15(2):e1002598 doi: 10.1371/journal.pbio.1002598 28234892 10.1371/journal.pbio.1002598 PMC5325181

[45] Kramer A, Erickson K. Capitalizing on cortical plasticity: influence of physical activity on cognition and brain function. Trends in Cognitive Sciences. 2007;11(8):342–348. doi: 10.1016/j.tics.2007.06.009 17629545 10.1016/j.tics.2007.06.009

[46] Laurin D, Verreault R, Lindsay J, MacPherson K, Rockwood K. Physical Activity and Risk of Cognitive Impairment and Dementia in Elderly Persons. Archives of Neurology. 2001;58(3). 10.1001/archneur.58.3.498 11255456

[47] Sibley B, Etnier J. The Relationship between Physical Activity and Cognition in Children: A Meta-Analysis. Pediatric Exercise Science. 2003;15(3):243–256.

[48] Westbrook A, Braver T. Cognitive effort: A neuroeconomic approach. Cognitive, Affective, & Behavioral Neuroscience. 2015;15(2):395–415. 10.3758/s13415-015-0334-y PMC4445645 25673005

[49] Massar S, Libedinsky C, Weiyan C, Huettel S, Chee M. Separate and overlapping brain areas encode subjective value during delay and effort discounting. NeuroImage. 2015;120:104–113. doi: 10.1016/j.neuroimage.2015.06.080 26163803 10.1016/j.neuroimage.2015.06.080

[50] Prevost C, Pessiglione M, Metereau E, Clery-Melin M, Dreher J. Separate Valuation Subsystems for Delay and Effort Decision Costs. Journal of Neuroscience. 2010;30(42):14080–14090. doi: 10.1523/JNEUROSCI.2752-10.2010 20962229 10.1523/JNEUROSCI.2752-10.2010 PMC6634773

[51] Malesza M, Ostaszewski P. Relations Between Cloninger’s Dimensions of Temperament and Steepness of Delay and Effort Discounting of Monetary Rewards. Psychological Reports. 2013;112(3):694–705. doi: 10.2466/09.14.PR0.112.3.694-705 24245065 10.2466/09.14.PR0.112.3.694-705

[52] Ghods-Sharifi S, Floresco SB. Differential effects on effort discounting induced by inactivations of the nucleus accumbens core or shell. Behavioral Neuroscience. 2010;124(2):179–91. doi: 10.1037/a0018932 20364878 10.1037/a0018932

[53] Daunizeau J, Adam V, Rigoux L. VBA: A Probabilistic Treatment of Nonlinear Models for Neurobiological and Behavioural Data. PLoS Computational Biology. 2014;10(1):e1003441 doi: 10.1371/journal.pcbi.1003441 24465198 10.1371/journal.pcbi.1003441 PMC3900378

[54] Rigoux L, Stephan K, Friston K, Daunizeau J. Bayesian model selection for group studies—Revisited. NeuroImage. 2014;84:971–985. doi: 10.1016/j.neuroimage.2013.08.065 24018303 10.1016/j.neuroimage.2013.08.065

[55] Penny W. Comparing Dynamic Causal Models using AIC, BIC and Free Energy. NeuroImage. 2012;59(1):319–330. doi: 10.1016/j.neuroimage.2011.07.039 21864690 10.1016/j.neuroimage.2011.07.039 PMC3200437

